# Acute gastrointestinal bleeding: A comparison between variceal and nonvariceal gastrointestinal bleeding

**DOI:** 10.1097/MD.0000000000031543

**Published:** 2022-11-11

**Authors:** Iulia Raţiu, Raluca Lupuşoru, Alina Popescu, Ioan Sporea, Adrian Goldiş, Mirela Dănilă, Bogdan Miuţescu, Tudor Moga, Andreea Barbulescu, Roxana Şirli

**Affiliations:** a Advanced Regional Research Center in Gastroenterology and Hepatology, Department VII: Internal Medicine II, Discipline of Gastroenterology and Hepatology, “Victor Babes” University of Medicine and Pharmacy Timisoara, Romania; b Center for Modeling Biological Systems and Data Analysis, Department of Functional Sciences, “Victor Babes” University of Medicine and Pharmacy Timisoara, Romania.

**Keywords:** mortality, nonvariceal bleeding, predictors, rebleeding, variceal bleeding

## Abstract

Acute upper gastrointestinal bleeding (UGIB) is a typical medical emergency, with an incidence of 84 to 160 cases per 100,000 individuals and a mortality rate of approximately 10%. This study aimed to identify all cases of UGIB hospitalized in a tertiary gastroenterology department, to identify possible predictive factors involved in rebleeding and mortality, potential associations between different elements and the severity of bleeding, and the differences between the upper digestive hemorrhage due to nonvariceal and variceal bleeding. This was an observational, retrospective study of patients with UGIB admitted to the tertiary Department of Gastroenterology between January 2013 and December 2020. A total of 1499 patients were enrolled in the study. One thousand four hundred and ninety-nine patients were hospitalized for 7 years with active upper digestive hemorrhage, 504 variceal bleeding, and 995 nonvariceal bleeding. When comparing variceal with nonvariceal bleeding, in nonvariceal bleeding, the mean age was higher, similar sex, higher mortality rate, higher rebleeding rate, and higher hemorrhagic shock rate. Endoscopy treatment was also performed more frequently in variceal bleeding than in nonvariceal bleeding. Severe anemia was found more frequently in patients with variceal bleeding. The mortality rate was 10% in the entire study group, which was not significantly different between the 2 batches. However, the rebleeding rate is higher in patients with variceal gastrointestinal bleeding.

## 1. Introduction

Acute upper gastrointestinal bleeding (UGIB) is a typical medical emergency, with an incidence of 84 to 160 cases per 100,000 individuals and a mortality rate of approximately 10%.^[1]^ Despite endoscopic therapies and pharmacological management development, UGIB is still associated with considerable mortality and morbidity rates and high medical expenses.^[2–5]^

UGIB remains a significant cause of hospital admission. Several risk factors have been studied to stratify patients according to the risk of complications, such as rebleeding or death, and to predict the need for rapid clinical intervention.^[6]^ This study aimed to identify all cases of UGIB hospitalized in a tertiary gastroenterology department, to identify possible predictive factors involved in rebleeding and mortality, potential associations between different elements and the severity of bleeding, and the differences between the upper digestive hemorrhage due to nonvariceal and variceal bleeding.

## 2. Materials and Methods

### 2.1. Methods

This was an observational, retrospective study of patients with UGIB admitted to the tertiary Department of Gastroenterology from January 2013 to December 2020.

Given the nature of tertiary medical institutions, most patients are referred from other medical institutions because of the severity of their disease. Thus, endoscopy was performed as soon as possible, and most patients underwent endoscopy within 6 hours of admission after the patient was stabilized hemodynamically. The final diagnosis of UGIB and the type of lesion were defined after upper digestive endoscopy.

For esophageal variceal grading, we used the Japanese 3-grade classification system.^[7]^ It involves scoring varices as grade 1 (small), straight small-caliber varices; grade 2 (medium), moderately enlarged, beady varices covering less than one-third of the lumen; and grade 3 (large), markedly enlarged, nodular or tumor-shaped varices occupying more than one-third of the lumen. For gastric varices, we used Sarin’s classification^[8]^: gastroesophageal varix type 1: Extension of esophageal varices along the lesser curve, Gastroesophageal varix type 2: Extension of esophageal varices along a great curve, isolated gastric varix type 1, and isolated gastric varix type 2: varices in the stomach or duodenum.

For nonvariceal gastrointestinal bleeding, lesions were described according to Forrest classification^[5]^: Forrest I a (Spurting hemorrhage); Forrest I b (Oozing hemorrhage); Forrest II a (non-bleeding visible vessel); Forrest II b (adherent clot); Forrest II c (flat pigmented hematin (coffee ground base) on ulcer base); Forrest III (lesions without signs of recent hemorrhage or fibrin-covered clean ulcer base). Comorbidities have been documented by clinical, paraclinical, and imaging examinations.

Treatment for nonvariceal gastrointestinal bleeding was divided into 2 groups: conservative, which did not require endoscopic therapy; and endoscopic therapy, divided into a single method (hemostatic clip or thermal coagulation), or combined endoscopic methods (adrenaline injection + clip or coagulation).

### 2.2. Study population

A total of 1499 patients were enrolled in the study. Socio-demographic data, clinical, paraclinical, and imaging examinations, type of lesion on endoscopy, therapeutic approaches, and response to treatment were recorded.

The inclusion criteria in the study were all patients with the upper gastrointestinal hemorrhage who underwent endoscopy. The exclusion criteria were hemorrhagic shock with death prior to endoscopy, absence of upper endoscopy, and lack of informed consent.

Clinical and biological recorded data included age, sex, medication history (non-steroidal anti-inflammatory drugs [NSAID] use, antiplatelet agents, and anticoagulants), personal history (liver cirrhosis, cardiovascular disease), systolic blood pressure, and heart rate at hospital admission. The shock was defined as systolic blood pressure < 100 mm Hg and heart rate > 100 beats/minute.

All patients suspected of having nonvariceal gastrointestinal bleeding received antisecretory treatment from admission, a bolus of 80 mg of Pantoprazole, and then 8 mg/hour for 3 days; All patients suspected with variceal bleeding were managed according to the Baveno VI consensus^[5]^ and received treatment with vasopressors (Terlipressin 1, 2 grams every 4 hours) and intravenous antibiotics. When the cause of bleeding was uncertain before endoscopy, both vasopressors and pump inhibitors were administered.

According to Romanian legislation, ethical committee approval was not required as a retrospective study. All patients provided informed consent for their data to be used for scientific purposes and for procedures to be made. This study was conducted by the Declaration of Helsinki.

### 2.3. Statistical analysis

Data are presented as mean (± standard deviation) for continuous variables with normal distribution, as average (interquartile range) for continuous variables without normal distribution, or as percentages (absolute frequencies) for nominal variables. The normality test was performed using the Kolmogorov-Smirnov test. Significant differences between groups were found using the t-test for normal distribution, the Mann-Whitney *U* for non-normal distribution, the Pearson chi-squared test, or the Fisher test for proportions. Statistical analysis of the data was performed using SPSS v.17 (SPSS Inc., Chicago, IL) and Microsoft Office Excel 2019. A *P*-value < .05 and a confidence interval of 0.05 were considered to be statistically significant.

## 3. Results

### 3.1. Patients characteristics

**Figure 1.**
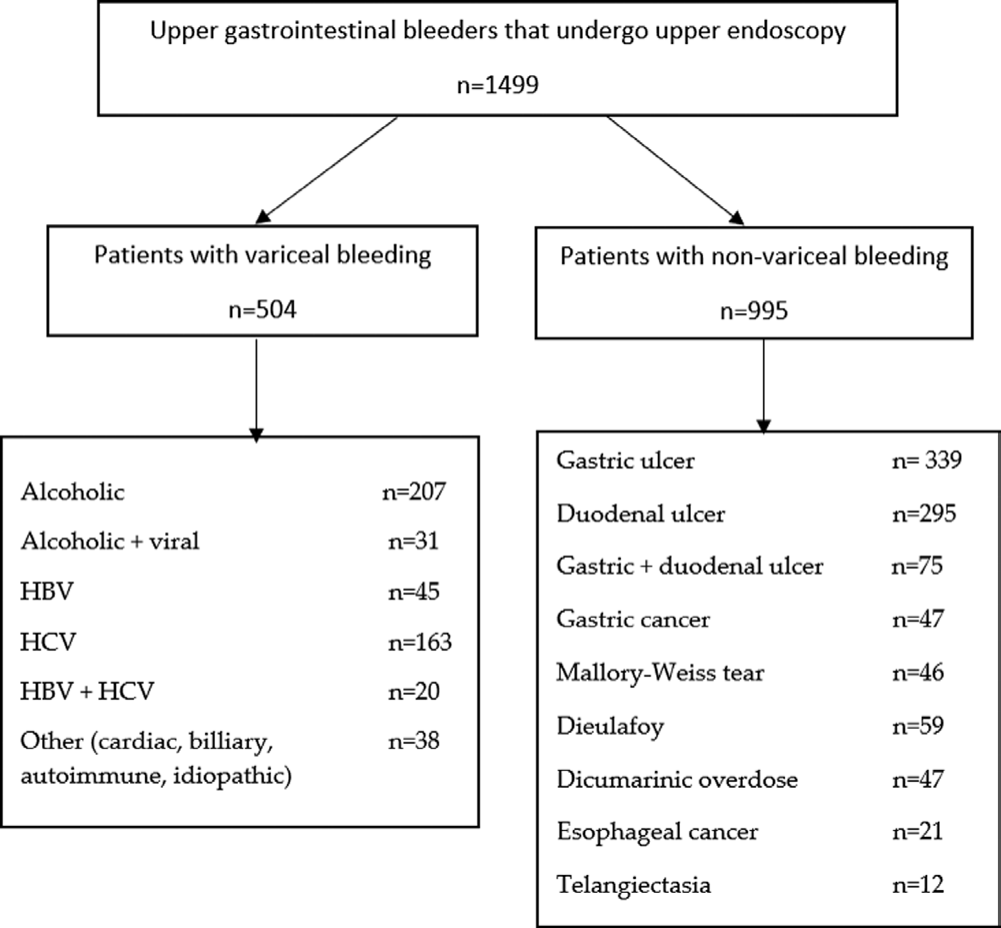
Study flowchart.

We analyzed the files of 1499 patients with UGIB. The endoscopic findings are shown in Figure 1. In 504/1499 (33.6%) cases, the UGIB was caused by variceal hemorrhage, while in 995/1499 (66.4%) cases, the hemorrhage was nonvariceal (*P* < .0001). The baseline characteristics of the study group and comparison between the nonvariceal and variceal groups are presented in Table 1.

**Table 1:** Comparison between the variceal and non-variceal study population.

Parameter	Variceal	Non-variceal	*P*-value
Age (years/mean ± SD)	59 ± 11.2	65 ± 14.9	<.0001
Gender (n, %)			
Female (n, %)	200 (36.6%)	338 (33.9%)	.08
Male (n, %)	304 (60.4%)	657 (66.1%)	.08
Rebleeding rate (n, %)	18.4%	8.8%	<.0001
Mortality rate (n, %)	8.5%	10.7%	.2
Hemorrhagic shock (n, %)	6.7%	9.8%	.05
Anemia			
Mild (n, %)	201 (40.1%)	227 (22.8%)	<.0001
Moderate (n, %)	136 (26.9%)	442 (44.4%)	<.0001
Severe (n, %)	146 (28.9%)	226 (22.8%)	.009
Total bilirubine (mg/dL, mean ± SD)	3.40 ± 1.86	1.03 ± 0.7	<.0001
Creatinine (mg/dL, mean ± SD)	1.12 ± 0.78	1.01 ± 0.1	<.0001
Thrombocytopenia (n, %)	420 (83.4%)	105 (10.5%)	<.0001
ALT (UI/L, mean ± SD)	59.0 ± 9.5	34.1 ± 8.4	<.0001
AST (UI/L, mean ± SD)	93.3 ± 52.0	40.1 ± 6.5	<.0001
INR (mean ± SD)	1.41 ± 0.5	1.02 ± 0.01	<.0001
Natrium (mmmol/L, mean ± SD)	135.8 ± 7.7	138.0 ± 5.2	<.0001
Potasium (mmol/L, mean ± SD)	3.7 ± 1.2	4.1 ± 0.9	<.0001
Surgery transfer/embolization (n, %)	2 (0.3%)	29 (2.9%)	.0007
Therapy			<.0001
PPI only (n, %)	–	439 (44.1%)	
Endoscopic therapy (n, %)	502 (99.6%)	556 (55.7%)	
Variceal banding (n, %)	472 (94.1%)	–	
Variceal sclerosis (n, %)	30 (5.9%)	–	
Single method(hemostatic clip/thermal coagulation)	–	296 (29.7%)	
Combined methods (adrenaline injection + clip/coagulation)	–	260 (26.2%)	
Presentation (n, %)			
Melena	203 (40.2%)	500 (50.2%)	.0002
Hematemesis	84 (8.4%)	60 (6.0%)	.08
Melena + hemetemesis	119 (32.0%)	184 (18.6%)	<.0001
Hematochesia	98 (19.4%)	251 (25.2%)	.01
Blood transfusion	144 (28.5%)	220 (22.1%)	.006
Hospitalization days	9.7 ± 1.2	5.0 ± 0.5	<.0001

ALT *=* alaniane aminotransferase, AST *=* aspartat aminotransferase; proton pump inhibitor, INR *=* international normalized ratio, SD *=* standard deviation.

### 3.2. Comparison between variceal and nonvariceal bleeding

When comparing variceal with nonvariceal bleeding, in nonvariceal bleeding, the mean age was higher, similar sex, higher mortality rate, higher rebleeding rate, and higher hemorrhagic shock rate. Endoscopy treatment was also performed more frequently in variceal bleeding than in nonvariceal bleeding. Severe anemia was found more frequently in patients with variceal bleeding (Table 1).

### 3.3. Nonvariceal bleeding

Most nonvariceal digestive hemorrhages could be managed conservatively (Table 1). Mortality and rebleeding rates were higher in the simple therapy group than in the combination therapy group (45% vs 23%, *P* < .0001; 25% vs 20%, *P* < .0001). NSAIDs, antiplatelet, and anticoagulant medications were significantly higher in patients over 60 years of age (68.5% vs 31.5%, *P* < .001). The highest incidence of nonvariceal bleeding was in the 71 to 80 years group (28.1%), which was significantly higher than the rest of the age groups (*P* = .02), with significantly higher consumption of NSAIDs and antiplatelet agents (*P* < .0001) (Table 2).

**Table 2:** Other baseline characteristics for non-variceal patients.

Parameter	n (%)
Forrest classification	
Ia	43 (6.0%)
Ib	103 (13.4%)
IIa	146 (19.1%)
IIb	105 (13.7%)
IIc	157 (20.5%)
III	209 (27.3%)
Susceptible factors	
Aspirin	184 (18.4%)
Other NSAID’s	145 (14.5%)
Antiplatelets	169 (16.8%)
Anticoagulants	160 (16.0%)
Helicobacter pylori	157 (15.7%)
Other	180 (18.6%)

NSAID = non-steroidal anti-inflamatory drugs.

Severe anemia doubles the risk of rebleeding (Odds ratio [OR] = 2; 95% Confidence interval [CI] 1.13–3.52). Severe anemia increased the risk of death in non-variceal hemorrhages by 4 times (OR = 4; 95% CI 2.1–6.75); hemorrhagic shock increased the risk of death by 3.4 times (OR = 3.4; 95% CI 1.34–6.5); antiplatelet agents, anticoagulants, and NSAIDs increased the risk of death by 3.5-, 2 and 1.7 times respectively (OR = 3.5; OR = 2; OR = 1.7) (Table 3).

**Table 3:** Predictive factors for rebleeding in nonvariceal hemorrhage.

Variable	Rebleeding (*P*-value)			Mortality (*P*-value)		
	OR	95% CI	*P*-value	OR	95% CI	*P*-value
Severe anemia	2.00	1.13–3.52	.001	4.00	2.10–6.75	<.0001
Age > 60	1.10	0.99–1.35	.02	1.15	0.99–1.89	.001
Aspirin	1.00	0.99–1.00	.08	1.00	0.99–1.15	.009
Anticoagulants	2.00	1.52–2.51	.87	1.08	1.00–1.90	.04
Antiplatelets	3.50	1.02–5.20	.90	1.00	0.89–1.15	.01
Other NSAID’s	1.70	1.10–3.40	.06	1.30	1.15–2.10	.01
Hemorrhagic shock	1.10	1.00–1.25	.05	3.40	1.34–6.50	<.0001

CI = confidence interval, NSAID = non-steroidal anti-inflatory drugs, OR = odds ratio.

Severe anemia (*P* = .01) was an independent predictive factor in the multivariate analysis of factors involved in rebleeding in nonvariceal hemorrhage. Severe anemia (*P* = .03) was an independent predictor of mortality.

### 3.4. Variceal bleeding

Baseline characteristics of variceal group are presented in Table 4. The presence of gastric varices increased the risk of rebleeding 3.3 times (OR = 3.3; 95% CI 1.96–5.63), the presence of grade III esophageal varices increased the risk of rebleeding by 2.25 times (OR = 2.25; 95% CI 1.36–3.72), severe anemia increased the risk of rebleeding 2 times (OR = 2; 95% CI 1.12–2.83), and age > 60 years increased the risk of bleeding 1.6 times (OR = 1.6; 95% CI 1.3–3.43). Severe anemia may increase the risk of death from upper variceal hemorrhage 19 times (OR = 19; 95% CI 7.6–23.5); Child-Pugh C class increased the mortality risk by 5.6 times (OR = 5.6; 95% CI 2.91–10.8) (Table 5).

**Table 4:** Other baseline characteristics of variceal bleeding patients.

Parameter	n (%)
Child-Pugh-Turcotte Classification	
A	182 (36.1%)
B	185 (36.7%)
C	137 (27.2%)
Esophageal varices (Japanese classification)	
Grade I	72 (14.2%)
Grade II	263 (52.1%)
Grade III	168 (33.7%)
Splenomegaly	429 (85.1%)
Gastric Varices	111 (22.2%)
Hepatic encefalopathy	123 (12.3%)
Ascites	299 (59.4%)

**Table 5:** Predictive factors for rebleeding and mortality in variceal bleeding.

Variable	Rebleeding			Mortality		
	OR	95% CI	*P*-value	OR	95% CI	*P*-value
Severe anemia	2.00	1.12–2.83	<.0001	19.0	7.60–23.5	<.0001
Moderate anemia	1.00	1.00–1.13	.03	1.00	1.00–1.89	.08
Age > 60	1.60	1.3–3.43	.001	1.60	0.85–2.96	.57
Esophageal varices gr II	1.01	1.00–1.89	.01	1.05	0.99–1.10	.06
Esophageal varices gr III	2.25	1.36–3.72	.001	2.20	1.25–4.20	.01
Gastric varices	3.30	1.96–5.63	<.0001	2.50	1.57–3.50	.05
Child-Pugh C	1.01	0.98–1.56	.04	5.60	2.91–10.80	<.0001
Hemorrhagic shock	1.00	1.00–2.45	.31	2.00	1.16–2.90	.04
Thrombocytopenia	1.00	1.01–3.21	.25	1.20	1.00–1.90	<.0001
Splenomegaly	1.01	1.00–4.52	.07	1.01	1.00–1.52	.02
Serum creatinine level	1.00	0.89–1.25	.12	1.01	1.00–1.65	.02
Encephalopathy	0.99	0.99–1.01	.89	1.15	0.99–2.14	0.01
Rebleeding	–	–	–	2.50	1.15–3.89	.04
TGP	0.98	0.75–1.01	.08	0.99	0.99–1.15	.0005
Hypoalbuminemia	1.10	1.00–2.14	.92	1.00	1.00–1.85	.001
Male gender	1.50	0.85–1.75	.25	1.00	0.98–1.56	.04

CI = confidence interval, OR = odds ratio.

In the multivariate analysis of factors involved in rebleeding in variceal hemorrhage, severe anemia (*P* = .006), Child-Pugh class C (*P* = .04), and gastric varices (*P* < .0001) were independent predictive factors. Factors involved in mortality in variceal bleeding, such as severe anemia (*P* = .01), age > 60 years (*P* = .001), thrombocytopenia (*P* < .0001), and Child-Pugh class C (*P* = .034) were independent predictors of mortality.

## 4. Discussion

This study aimed to identify the epidemiological characteristics and prognostic factors of upper GI bleeding. The mean age was 61 years, with approximately 70% of patients over 60 years of age. As in the literature, the most frequently encountered cases of upper digestive hemorrhage were nonvariceal, and among them, the most common were gastroduodenal ulcers.^[9,10]^ In our study, the proportion of nonvariceal bleeders was higher than that of variceal bleeders, but the fact that it is a tertiary referral center should give us the opposite, with a higher proportion of variceal bleeders, as in the paper published by Rout et al^[11]^

The mortality rate was approximately 10% in the entire batch, a rate similar to that reported in other European studies^[12,13]^: 10.7% in patients with nonvariceal UGIB and 8.5% in those with variceal UGIB, higher in the nonvariceal bleeding group. The rebleeding rate was higher in the variceal group, similar to the literature.^[14]^

In the group with nonvariceal hemorrhage, the mean age was higher, and hemorrhagic shock was more frequent than in the variceal hemorrhage group. Severe anemia was more frequent in the variceal hemorrhage group. The bioclinical constans were more likely to be normal in the nonvariceal group.

Regarding therapy, endoscopic methods were used more frequently in the variceal bleeding group because all efracted varices were treated. 44.1% of patients received only proton pump inhibitors in the nonvariceal group and did not need any endoscopic treatment.

Most of the patients presented at admission with melena in both groups, but a higher rate in nonvariceal bleeders, while hematemesis had a higher rate in the variceal bleeding group. Blood transfusion was given more in the variceal bleeding group. The mean number of hospitalization days was higher for variceal bleeders. A fact can be their condition, with decompensated liver cirrhosis.

Most studies have focused on identifying the risk factors and unfavorable prognosis of gastrointestinal bleeding. These factors are essential to remember because the entire management of the patient may depend on them, or, more importantly, placing them in advance can prevent a bleeding episode. Many risk factors are known to influence outcomes in UGIB settings. Age, comorbidities, hypovolemic shock, endoscopic diagnosis, hemoglobin values at the time, ulcer size, stigmata of recent hemorrhage, and need for a blood transfusion have all been described as significant risk factors for rebleeding and death.^[12,15–17]^ Severe anemia and age over 60 years were predictors of rebleeding in variceal and nonvariceal bleeding groups.

The widespread use of potent PPIs and treatment of Helicobacter pylori should decrease the incidence of nonvariceal UGI bleeding; however, in recent years, there has been an increasing proportion of elderly patients^[18]^ with the presence of multiple comorbidities, leading to increased consumption of NSAIDs and antiplatelet treatment. In this context, nonvariceal hemorrhage continues to be associated with significant morbidity and mortality. In the nonvariceal bleeding group, the consumption of anticoagulants, antiplatelets, NSAIDs, and aspirin were predictive factors for mortality. Many studies have compared various scoring systems as risk stratification methods.^[19]^ Patients with a low score usually do not need endoscopic hemostasis, and elective endoscopy can be scheduled as an outpatient later.^[20]^ In contrast, a high-risk score is associated with a high blood transfusion rate, the need for endoscopic therapy, and prolonged hospitalization.^[21,22]^ In our study, severe anemia (*P* = .03) was an independent predictor of mortality.

This study found that the rate of in-hospital rebleeding and mortality in variceal hemorrhage batches could be as high as 18.4% and 8.5%, respectively, and the mortality rate was higher in patients with in-hospital rebleeding than in those without. These findings are similar to those reported in previous studies.^[23,24]^ Even if the rebleeng rate was higher in the variceal group, the mortality rate was higher in the non-variceal group. This fact is in concordance with the literature,^[23]^ and may be due to the greater hemorrhage of the big ulcers, that can be fatal.

Randomised controlled trials have shown that mortality due to variceal bleeding in cirrhosis has decreased over the past decades but is still remarkably high. Hence, stratifying the risk of mortality is paramount.^[25]^ The best method for stratifying risk is unclear. The variables analyzed in previous studies as possible prognostic factors were age, systolic blood pressure, heart rate, hemoglobin, comorbidity, albumin, international normalized ratio, and blood urea nitrogen. Previous studies have found that these variables are independently associated with short-term mortality in patients with cirrhosis.^[26,27]^ Amitrano et al^[28]^ concluded that CTP class C was an independent predictor of 5-days failure and Bambha et al^[29]^ demonstrated that patients who received ≥ 4 units of packed erythrocytes within the first 24 hours or were actively bleeding at the time of endoscopy had an increased mortality rate. In our study group, esophageal varices grade III, Child Pugh class C, and hemorrhagic shock were predictors of both rebleeding and mortality. The presence of gastric varices increases the risk of rebleeding 3.3 times. Factors involved in mortality in variceal bleeding, such as severe anemia (*P* = .01), age > 60 years (*P* = .001), thrombocytopenia (*P* < .0001), and Child-Pugh class C (*P* = .034) were independent predictors of mortality.

A strong point of our study was that a substantial cohort was used, the power of the study was 80%, and all patients underwent upper digestive endoscopy; however, the study has some limitations: data collection was retrospective, and it was a single-center study, implying potential data bias. However, because the Gastroenterology Department represents a tertiary referral center covering the country’s western region, the emergency unit receives many critical cases with UGIB from all neighboring counties. Second, this observational study focused on high-risk mortality factors and classified high-risk patients. However, the criteria for low-risk patients were not established, and neither the requirements for the need for endoscopic treatment nor those for outpatient follow-up were presented. There was no follow-up of patients in terms of out-of-hospital mortality, only the in-hospital mortality rate was followed, and all causes of death, not only those related to bleeding episodes, were included.

## 5. Conclusions

One thousand four hundred and ninety-nine patients were hospitalized for 7 years with active upper digestive hemorrhage, 504 variceal bleeding, and 995 nonvariceal bleeding. The mortality rate was 10% in the entire study group, which was not significantly different between the 2 batches. However, the rebleeding rate is higher in patients with variceal gastrointestinal bleeding.

## Author contributions

**Conceptualization:** Iulia Raţiu, Raluca Lupuşoru.

**Data curation:** Iulia Raţiu, Raluca Lupuşoru, Andreea Barbulescu.

**Formal analysis:** Raluca Lupuşoru.

**Investigation:** Iulia Raţiu, Raluca Lupuşoru, Bogdan Miuţescu, Tudor Moga, Andreea Barbulescu, Roxana Şirli, Mirela Dănilă.

**Methodology:** Iulia Raţiu, Raluca Lupuşoru.

**Project administration:** Iulia Raţiu, Alina Popescu, Ioan Sporea.

**Resources:** Mirela Dănilă, Ioan Sporea.

**Software:** Raluca Lupuşoru.

**Supervision:** Alina Popescu, Ioan Sporea.

**Validation:** Iulia Raţiu, Raluca Lupuşoru, Alina Popescu, Ioan Sporea, Adrian Goldiş.

**Visualization:** Iulia Raţiu.

**Writing – original draft:** Iulia Raţiu, Raluca Lupuşoru, Roxana Şirli.

**Writing – review & editing:** Iulia Raţiu, Raluca Lupuşoru, Alina Popescu, Ioan Sporea, Adrian Goldiş, Mirela Dănilă, Bogdan Miuţescu, Tudor Moga, Roxana Şirli.

## References

[1] Kurien M Lobo A. Acute upper gastrointestinal bleeding. Clin Med (Lond). 2015;15:481–5. 26430191 10.7861/clinmedicine.15-5-481 PMC4953237

[2] Abougergi MS Travis AC Saltzman JR. The in-hospital mortality rate for upper GI hemorrhage has decreased over 2 decades in the United States: a nationwide analysis. Gastrointest Endosc. 2015;81:882–8.e1. 25484324 10.1016/j.gie.2014.09.027

[3] Leontiadis GI Molloy-Bland M Moayyedi P. Effect of comorbidity on mortality in patients with peptic ulcer bleeding: systematic review and meta-analysis. Am J Gastroenterol. 2013;108:331–45; quiz 346. 23381016 10.1038/ajg.2012.451

[4] Sey MSL Mohammed SB Brahmania M. Comparative outcomes in patients with ulcer- vs non-ulcer-related acute upper gastrointestinal bleeding in the United Kingdom: a nationwide cohort of 4474 patients. Aliment Pharmacol Ther. 2019;49:537–45. 30628112 10.1111/apt.15092

[5] Gralnek IM Dumonceau JM Kuipers EJ. diagnosis and management of nonvariceal upper gastrointestinal hemorrhage: European society of gastrointestinal endoscopy (ESGE) guideline. Endoscopy. 2015;47:a1–46. 26417980 10.1055/s-0034-1393172

[6] Monteiro S Gonçalves TC Magalhães J. Upper gastrointestinal bleeding risk scores: who, when and why? World J Gastrointest Pathophysiol. 2016;7:86–96. 26909231 10.4291/wjgp.v7.i1.86 PMC4753192

[7] Tajiri T Yoshida H Obara K. General rules for recording endoscopic findings of esophagogastric varices (2nd edition). Dig Endosc. 2010;22:1–9. 20078657 10.1111/j.1443-1661.2009.00929.x

[8] Sarin SK Lahoti D Saxena SP. Prevalence, classification and natural history of gastric varices: a long-term follow-up study in 568 portal hypertension patients. Hepatology. 1992;16:1343–9. 1446890 10.1002/hep.1840160607

[9] Wuerth BA Rockey DC. Changing epidemiology of upper gastrointestinal hemorrhage in the last decade: a nationwide analysis. Dig Dis Sci. 2018;63:1286–93. 29282637 10.1007/s10620-017-4882-6

[10] Hearnshaw SA Logan RFA Lowe D. Acute upper gastrointestinal bleeding in the UK: patient characteristics, diagnoses and outcomes in the 2007 UK audit. Gut. 2011;60:1327–35. 21490373 10.1136/gut.2010.228437

[11] Rout G Sharma S Gunjan D. Comparison of various prognostic scores in variceal and nonvariceal upper gastrointestinal bleeding: a prospective cohort study. Indian J Gastroenterol. 2019;38:158–66. 30830583 10.1007/s12664-018-0928-8

[12] Nahon S Hagège H Latrive JP. Epidemiological and prognostic factors involved in upper gastrointestinal bleeding: results of a French prospective multicenter study. Endoscopy. 2012;44:998–1008. 23108771 10.1055/s-0032-1310006

[13] Chiu PW Ng EK. Predicting poor outcome from acute upper gastrointestinal hemorrhage. Gastroenterol Clin North Am. 2009;38:215–30. 19446255 10.1016/j.gtc.2009.03.009

[14] Tandon P Bishay K Fisher S. Comparison of clinical outcomes between variceal and nonvariceal gastrointestinal bleeding in patients with cirrhosis. J Gastroenterol Hepatol. 2018;33:1773–9. 29601652 10.1111/jgh.14147

[15] Klebl F Bregenzer N Schöfer L. Risk factors for mortality in severe upper gastrointestinal bleeding. Int J Colorectal Dis. 2005;20:49–56. 15322836 10.1007/s00384-004-0624-2

[16] Zimmerman J Siguencia J Tsvang E. Predictors of mortality in patients admitted to hospital for acute upper gastrointestinal hemorrhage. Scand J Gastroenterol. 1995;30:327–31. 7610347 10.3109/00365529509093285

[17] Imperiale TF Dominitz JA Provenzale DT. Predicting poor outcome from acute upper gastrointestinal hemorrhage. Arch Intern Med. 2007;167:1291–6. 17592103 10.1001/archinte.167.12.1291

[18] Yachimski PS Friedman L. Gastrointestinal bleeding in the elderly. Nat Clin Pract Gastroenterol Hepatol. 2008;5:80–93. 18253137 10.1038/ncpgasthep1034

[19] Kim BJ Park MK Kim SJ. Comparison of scoring systems for the prediction of outcomes in patients with nonvariceal upper gastrointestinal bleeding: a prospective study. Dig Dis Sci. 2009;54:2523–9. 19104934 10.1007/s10620-008-0654-7

[20] Le Jeune IR Gordon AL Farrugia D. Safe discharge of patients with low-risk upper gastrointestinal bleeding (UGIB): can the use of glasgow-blatchford bleeding score be extended? Acute Med. 2011;10:176–81. 22111089

[21] Shrestha UK Sapkota S. Etiology and adverse outcome predictors of upper gastrointestinal bleeding in 589 patients in Nepal. Dig Dis Sci. 2014; 59:814–22. 24282053 10.1007/s10620-013-2946-9

[22] Lazăr DC Ursoniu S Goldiş A. Predictors of rebleeding and in-hospital mortality in patients with nonvariceal upper digestive bleeding. World J Clin Cases. 2019;7:2687–703. 31616685 10.12998/wjcc.v7.i18.2687 PMC6789381

[23] Jairath V Rehal S Logan R. Acute variceal haemorrhage in the United Kingdom: patient characteristics, management and outcomes in a nationwide audit. Dig Liver Dis. 2014;46:419–26. 24433997 10.1016/j.dld.2013.12.010

[24] Sanders DS Carter MJ Goodchap RJ. Prospective validation of the Rockall risk scoring system for upper GI hemorrhage in subgroups of patients with varices and peptic ulcers. Am J Gastroenterol. 2002;97:630–5. 11922558 10.1111/j.1572-0241.2002.05541.x

[25] Fortune BE Garcia-Tsao G Ciarleglio M. Child-turcotte-pugh class is best at stratifying risk in variceal hemorrhage: analysis of a US multicenter prospective study. J Clin Gastroenterol. 2017;51:446–53. 27779613 10.1097/MCG.0000000000000733 PMC5403609

[26] Krige JE Kotze UK Distiller G. Predictive factors for rebleeding and death in alcoholic cirrhotic patients with acute variceal bleeding: a multivariate analysis. World J Surg. 2009;33:2127–35. 19672651 10.1007/s00268-009-0172-6

[27] Jepsen P. Comorbidity in cirrhosis. World J Gastroenterol. 2014;20:7223–30. 24966593 10.3748/wjg.v20.i23.7223 PMC4064068

[28] Amitrano L Guardascione MA Manguso F. The effectiveness of current acute variceal bleed treatments in unselected cirrhotic patients: refining short-term prognosis and risk factors. Am J Gastroenterol. 2012;107:1872–8. 23007003 10.1038/ajg.2012.313

[29] Bambha K Kim WR Pedersen R. Predictors of early rebleeding and mortality after acute variceal haemorrhage in patients with cirrhosis. Gut. 2008;57:814–20. 18250126 10.1136/gut.2007.137489

